# Krankenhausalarm- und -einsatzplanung in Baden-Württemberg. Eine länderspezifische Umfrage an 214 Kliniken

**DOI:** 10.1007/s10049-022-01065-1

**Published:** 2022-08-16

**Authors:** Ernst G. Pfenninger, Sabine Villhauer, Manuel Königsdorfer

**Affiliations:** 1 Stabsstelle Katastrophenschutz, Universitätsklinikum Ulm, Albert-Einstein-Allee 29, 89081 Ulm, Deutschland; 2 Klinik für Anästhesiologie und Intensivmedizin, Universitätsklinikum Ulm, Ulm, Deutschland

**Keywords:** Katastrophenschutz, Umfrage, Kliniknotfallpläne, Kritische Infrastruktur, Klinische Vorbereitung, Disaster response, Survey, Hospital disaster drills, Critical infrastructure, Clinical preparedness

## Abstract

**Hintergrund:**

Öffentlich geförderte Akutkrankenhäuser wirken in Deutschland aufgrund gesetzlicher Vorgaben im Katastrophenschutz mit. Diese Mitwirkung umfasst insbesondere auch die Notwendigkeit, Alarm- und Einsatzpläne für interne und externe Gefahrenlagen aufzustellen und fortzuschreiben und sich auf Anforderung der Behörden an Übungen zu beteiligen. Literatur, ob und in welchem Umfang die Krankenhäuser diesen Verpflichtungen nachkommen, ist bisher jedoch nur sehr eingeschränkt verfügbar.

**Fragestellung:**

In einem standardisierten Abfrageverfahren sollte der aktuelle Status der Alarm- und Einsatzplanung in baden-württembergischen Krankenhäusern evaluiert werden.

**Material und Methoden:**

An 214 anhand einer Auflistung der Baden-Württembergischen Krankenhausgesellschaft e. V. (BWKG) identifizierten Kliniken in Baden-Württemberg wurde ein einheitlicher Fragebogen versendet, in dem bestimmte Merkmale des Alarm- und Einsatzplans, dessen Verfügbarkeit, die regelmäßige Beteiligung der Klinik an Katastrophenschutzübungen sowie Folgerungen daraus abgefragt wurden.

**Ergebnisse:**

Von den 214 Kliniken in Baden-Württemberg gaben 135 (63 %) Rückmeldung anhand des Fragebogens. Die Alarm- und Einsatzpläne enthalten in 79,3 % sowohl externe (z. B. Massenanfall von Verletzten) als auch interne Gefahrenlagen (z. B. Brände, Ausfall technischer Anlagen). Im weit überwiegenden Anteil der Fälle (94 %) gaben die Kliniken an, den Notfallplan regelmäßig zu aktualisieren, das Zeitintervall der Aktualisierung variiert jedoch stark. Drei Viertel der Krankenhäuser führen regelmäßig Teil- oder Vollübungen durch. Teilweise fanden die aus den Übungen gewonnenen Erkenntnisse Einzug in den Alarm- und Einsatzplan oder führten zur verbesserten Schulung von Mitarbeitenden.

**Schlussfolgerung:**

Die Bereitschaft der Krankenhäuser, eine umfassende Notfallplanung aufzustellen und sich an entsprechenden Übungen zu beteiligen, hat in den vergangenen Jahren merklich zugenommen. Weiterhin besteht jedoch in manchen Kliniken ein Mangel bei der Aktualisierungsfrequenz der Alarm- und Einsatzpläne. Bei kleineren Kliniken bestehen zudem noch Defizite in Bezug auf vorbereitende Maßnahmen gegen interne Gefahrenlagen, die aus dem Ausfall technischer Anlagen resultieren. Vermehrt sollten regelmäßige Übungen durchgeführt werden, um die festgelegten Verfahren auf den Prüfstand zu stellen und die Beschäftigten mit den Abläufen routinemäßig vertraut zu machen.

**Zusatzmaterial online:**

Die Online-Version dieses Beitrags (10.1007/s10049-022-01065-1) enthält den Fragebogen zum Thema Katastrophenplan.

## Abstract (en)

**Background:**

Due to legal regulations in Germany, public acute and emergency (A&E) hospitals—along with responsible authorities, emergency medical services, and other institutions such as the state medical associations—are committed to participate in civil protection. This participation includes the need to create and update emergency plans for external and internal crises and to take part in disaster drills. In fact, so far there is only little literature to prove whether and to what extent hospitals fulfill their obligations on this topic

**Objectives:**

Using a standardized survey, the state of emergency planning in hospitals in Baden-Wuerttemberg was evaluated.

**Materials and methods:**

Based on a listing provided by the Hospital Society of Baden-Wuerttemberg (BWKG), all 214 hospitals in Baden-Wuerttemberg were identified. The standardized questionnaire inquired about specific characteristics of the emergency plan, the availability and knowledge of this plan by the hospital workforce and other local institutions that take part in civil protection and, finally, participation in disaster drills were queried.

**Results:**

Of the 214 hospitals in Baden-Wuerttemberg, 135 (63%) provided information using the questionnaire. Except for one hospital, all other clinics indicated having a special emergency plan ready. In most cases (79.3%), both external (e.g., mass casualty incidents) and internal (e.g., fire, failure of technical equipment) crises are covered. In the vast majority of cases (94%), the hospitals also indicated that they regularly update their emergency plan, whereby the frequency of updates varied markedly. Three quarters of the hospitals said that they also regularly simulate the use of the emergency plan in disaster drills. In two thirds of the cases, external forces such as emergency medical services or the fire department also take part in these drills along with the hospitals themselves. In some cases, knowledge gained from the drills was incorporated into the emergency plan or led to improvements in staff training.

**Conclusions:**

The willingness of public hospitals to establish comprehensive disaster planning and to take part in related drills seems to have improved noticeably in recent years. However, there is still the need for improvement in keeping the concepts up to date at some hospitals. Especially smaller hospitals showed deficits in emergency planning, particularly concerning preparedness for internal crises, resulting from failure of technical equipment. More regular drills should be used to test existing concepts and to familiarize employees with the processes on a routine basis.

## Einleitung

Krankenhäuser zählen per definitionem zu den kritischen Infrastrukturen, die in Krisensituationen wichtige Bedeutung für das staatliche Gemeinwesen haben und bei deren Ausfall oder Beeinträchtigung nachhaltig wirkende Versorgungsengpässe, erhebliche Störungen der öffentlichen Sicherheit oder andere dramatische Folgen eintreten würden [1]. Massenanfall von Verletzten (MANV; [2]), Terroranschläge [3] oder Brandkatastrophen [4] stellen Kliniken dabei vor enorme personelle und organisatorische Herausforderungen. Moderne Krankenhäuser sind hochkomplexe technische Einrichtungen, deren Ausfall oder gezielte Störung Personal und Patienten gefährden kann [5]. Damit Kliniken bei von außen an sie herangetragenen oder intern entstandenen Gefahren- oder Schadenslagen nicht existenziell bedroht werden, kommt einer allumfassenden Notfallplanung, auch Krankenhausalarm- und -einsatzplanung genannt [6], enorme Bedeutung zu [7].

Nach den Vorgaben der Landeskatastrophenschutzgesetze in Deutschland haben öffentlich geförderte Akutkrankenhäuser und ihre Träger im Rahmen ihres Aufgabenbereichs im Katastrophenschutz mitzuwirken und sind verpflichtet, Alarm- und Einsatzpläne auszuarbeiten und weiterzuführen [8]. Allerdings sind in der Literatur über die tatsächliche Erfüllung dieser gesetzlichen Verpflichtung kaum Daten zu finden. Konzepte zu KAEP wurden in den letzten Jahren vielfach entworfen und auch diskutiert [9–13], das Bundesamt für Bevölkerungsschutz und Katastrophenhilfe (BBK) veröffentlichte im November 2020 zuletzt ein Handbuch zur „Krankenhausalarm- und -einsatzplanung“ [6], wegen weitgehend fehlender Erhebung aber wurden die realen Planungen an Kliniken kaum evaluiert. 1998 beklagten Autoren nach Analyse von über 500 Katastrophenschutzplänen, dass Kliniken nicht genügend auf Schadensereignisse vorbereitet sind [10]. Im deutschsprachigen Raum ergab eine Literatursuche eine Publikation im Jahr 2004 mit Ergebnissen hierzu [15] sowie eine Inauguraldissertation und deren Publikation in 2013 [16, 17]. Dabei wird die Notwendigkeit einer suffizienten KAEP in der Literatur vielfach betont [11–13]. Vergangene Schadenslagen in Kliniken haben jedoch gezeigt, dass diese entweder gar nicht oder nur mangelhaft darauf vorbereitet waren [12, 13].

In der vorliegenden Studie sollten länderspezifisch die Kliniken in Baden-Württemberg zu ihren Alarm- und Einsatzplänen, deren Ausgestaltung sowie darauf basierenden Übungen befragt werden. Die häufig verwendeten synonymen Bezeichnungen Katastrophen‑, Notfall‑, Evakuierungs- oder Alarmplan sowie Krankenhausalarm- und -einsatzplan (KAEP) werden im Folgenden einheitlich als KAEP zusammengefasst.

## Material und Methodik

Zur Datenerhebung wurde von den Autoren ein Fragebogen mit 17 Fragen konzipiert (siehe Online-Zusatzmaterial), wobei die ersten zwei Fragen zur Charakterisierung der Kliniken dienen. Im Hauptteil des Fragebogens wird nach Charakteristiken des KAEP sowie dessen Verfügbarkeit gefragt. Die letzten vier Fragen dienten der Eruierung von durchgeführten Übungen sowie den Folgerungen daraus. Die Mehrzahl der Fragen war so formuliert, dass nur eine Antwortmöglichkeit zulässig war, bei sechs Fragen waren Mehrfachnennungen möglich.

Am 1. September 2019 wurde der Fragebogen an alle 214 Kliniken in Baden-Württemberg verschickt mit der Bitte den ausgefüllten Bogen in anonymisierter Form an die Stabsstelle Katastrophenschutz des Universitätsklinikums Ulm zurückzusenden. Das Anschreiben erfolgte jeweils an die Verwaltungsdirektoren bzw. ärztlichen Direktoren der Kliniken. Nach 6 Wochen wurde an alle Kliniken ein Erinnerungsschreiben versandt und die Befragung am 15. November 2019 abgeschlossen. Das Klinikverzeichnis sowie die entsprechenden Anschriften wurden von der Baden-Württembergischen Krankenhausgesellschaft e. V. (BWKG) zur Verfügung gestellt.

Die Daten der retournierten Fragebögen wurden in eine Excel-Tabelle (Microsoft Corporation, Redmond, USA) übertragen und die weiteren deskriptiven statistischen Auswertungen dort durchgeführt. Die Ergebnisse wurden mittels absoluter und relativer Häufigkeit dargestellt. Die Prozentzahlen beziehen sich jeweils auf die Anzahl zurückgesendeter Fragebögen. Mit dem Chi-Quadrat-Test („Origin Pro 2017, Graphing & Analysis“, Origin Lab Corporation®, Northampton, MA, USA) wurden die Daten auf statistisch absicherbare Unterschiede geprüft. Ein *p*-Wert von weniger als 0,05 wird als signifikanter Unterschied angesehen.

## Ergebnisse

### Demografische Angaben

Von den 214 angeschriebenen Kliniken antworteten 135, das entspricht einer Rückmeldequote von über 63 %. Kliniken unter 300 Betten beteiligten sich hingegen an der Umfrage nur zu knapp 44 % (Tab. 1). Eine Klinik gab eine gemischte staatlich-kirchliche Trägerschaft an. Kliniken mit staatlicher Trägerschaft antworteten in 62,4 %, mit privatem Träger in 84,7 % und mit kirchlicher Trägerschaft in 38,9 %. Alle bis auf eine Klinik gaben an, dass sie über einen KAEP verfügen. Dieser ist in 74 % im Intranet für alle Beschäftigten verfügbar, liegt in 33 % in ausgedruckter Form vor, und ist damit jedem Mitarbeitenden zugänglich, und ist in 31 % in Schriftform nur in Sekretariaten oder Abteilungen vorhanden.

**Table 1:** 

Anzahl Betten	Angeschriebene Kliniken	Rücksendungen	Prozent
Unter 300	139	61	43,9
300–499	36	36	100,0
500–1000	23	23	100,0
Mehr als 1000	16	15	93,8
Gesamt	214	135	63,1
Ohne unter 300	–	–	97,9

### Charakterisierung der Alarm- und Einsatzpläne

**Figure 1:**
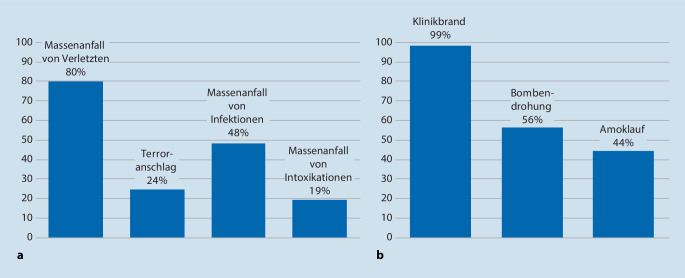

107 (79,3 %) der Antwortenden geben an, dass im KAEP externe und interne Gefahrenlagen behandelt werden, 26 unterscheiden nicht zwischen verschiedenen Notfallszenarien, 2 Antwortende machten keine Angabe. Abb. 1 zeigt die in den Alarm- und Einsatzplänen abgehandelten Szenarien, wobei der Massenanfall an Verletzten (MANV) sowie der Klinikbrand signifikant am häufigsten zu finden sind.

Nicht nur ein Klinikbrand, eine Bombendrohung oder ein Amoklauf kann fatale Auswirkungen auf ein Krankenhaus haben, sondern auch vielfältige technische Ausfälle. 81 % der Antwortenden gaben an, dass sie über einen Notfallplan für einen Stromausfall verfügen, 67 % sind auf einen EDV-Ausfall vorbereitet, 61 % auf einen Wasserausfall, 47 % auf einen Heizungs- bzw. Klimaanlagenausfall und 37 % auf einen Sauerstoffausfall. Vor allem Kliniken mit mehr als 500 bzw. 1000 Betten sind auf technische Probleme gut vorbereitet, so sind dies für einen Wasserausfall 78 % bzw. 73 %, wohingegen Kliniken mit weniger als 300 Betten hierfür nur in 51 % vorbereitet sind. Am wenigsten sind Kliniken auf einen Sauerstoffausfall vorbereitet, Kliniken mit mehr als 500 bzw. 1000 Betten zu 39 % bzw. 53 % und Kliniken unter 300 Betten nur zu 38 %. Am besten vorbereitet sind Krankenhäuser auf einen Stromausfall, hier 74 % der Kliniken unter 300 Betten bis hin zu 93 % bei Kliniken mit mehr als 1000 Betten.

Die Antwortenden geben an, dass in 94 % der Kliniken der KAEP regelmäßig aktualisiert werde, in 6 % ist dies nicht der Fall. Die Aktualisierungsintervalle zeigen sich jedoch interindividuell sehr variabel, wobei sich bezogen auf die Bettenanzahl keine signifikanten Unterschiede ergeben (Tab. 2). Auffällig ist, dass die Rettungsleitstelle nur in 71 % Kenntnis vom KAEP hat, in 27 % ist dies nicht der Fall, 2 % machten hierzu keine Angaben. Die Bekanntgabe beim örtlichen Rettungsdienst war bei den Kliniken unter 300 Betten mit 71 % am geringsten, um bei Kliniken über 1000 Betten auf 87 % anzusteigen.

**Table 2:** 

Wann wurde der Katastrophenplan zuletzt aktualisiert?					
Betten	Unter 300	300–499	500–1000	Über 1000	Gesamt
< 1 Jahr	35 (57 %)	16 (44 %)	12 (52 %)	7 (47 %)	70 (51,9 %)
Vor 1–2 Jahren	18 (30 %)	12 (33 %)	10 (44 %)	7 (47 %)	47 (34,8 %)
Vor 3–5 Jahren	4 (7 %)	6 (17 %)	0 (0 %)	1 (7 %)	11 (8,1 %)
Vor > 5 Jahren	2 (3 %)	2 (6 %)	1 (4 %)	0 (0 %)	5 (3,7 %)
Keine Angaben	2 (3 %)	0 (0 %)	0 (0 %)	0 (0 %)	2 (1,5 %)

### Übungen zu Gefahren- und Schadenslagen

**Figure 2:**
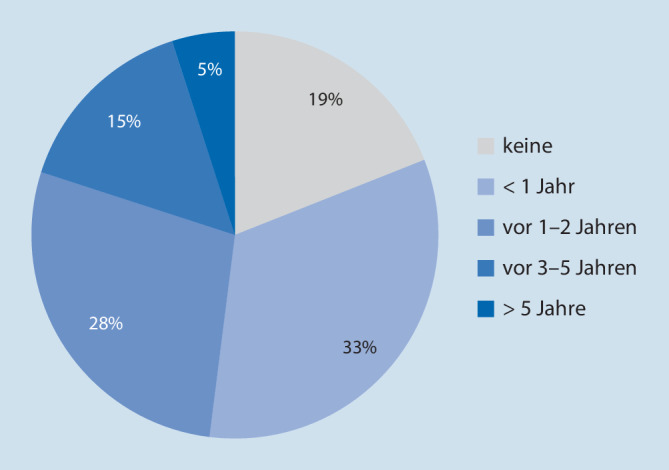

Übungen zur Gefahren- und Schadenslagen führen 75 % der Kliniken durch, 25 % hingegen nicht. Es handelt sich dabei zum Großteil um Teilübungen, mit 29 % eher selten um Vollübungen. Stabsübungen wurden in 37 % durchgeführt (Tab. 3). Die Kliniken geben in 94 % der Fälle an, dass sie eine personell definierte Einsatzleitung haben. Die Übungen wurden vorwiegend in einem Zeitraum weniger als ein Jahr zurückliegend durchgeführt. 19 % führten im angesprochenen Zeitraum keine Übungen durch (Abb. 2). Auf die Frage, ob bei den Übungen auch verschiedene Szenarien beübt wurden, antworteten 55 % mit Ja, 24 % mit Nein und 21 % machten keine Angaben. Bei Kliniken mit weniger als 300 Betten war hier die Verneinung bzw. die Enthaltung mit 10 % bzw. 15 % am höchsten, wohingegen nur 3 von 32 Kliniken mit mehr als 1000 Betten keine unterschiedlichen Szenarien beübten. Bei 62 % der übenden Kliniken waren in die Übungen auch externe Kräfte (z. B. Rettungsdienst, Feuerwehr, Polizei, ABC-Züge) eingebunden. Überraschenderweise war dies bei Klinken mit weniger als 300 Betten in 21 % der Fall, wohingegen in Kliniken mit mehr als 500 bzw. 1000 Betten dies nur in 13 % bzw. 10 % stattfand.

**Table 3:** 

Übungen zu Gefahren- und Schadenslagen					
Betten	Unter 300	300–499	500–1000	Über 1000	Gesamt
*Nein*	23^a^ (17 %)	7 (5 %)	3 (2 %)	0 (0 %)	33 (25 %)
*Ja*	37 (28 %)	29 (22 %)	20 (15 %)	15 (11 %)	100 (75 %)
*Stabsübung*	9 %	10 %	10 %	8 %	37 %
*Teilübung*	24 %	18 %	15 %	11 %	68 %
*Vollübung*	8 %	8 %	6 %	7 %	29 %

^a^„Unter 300“: signifikant weniger Übungen gegenüber Kliniken mit mehr als 500 bzw. 1000 Betten. Zwei Antwortende machten keine Angaben

### Folgerungen aus durchgeführten Übungen

**Figure 3:**
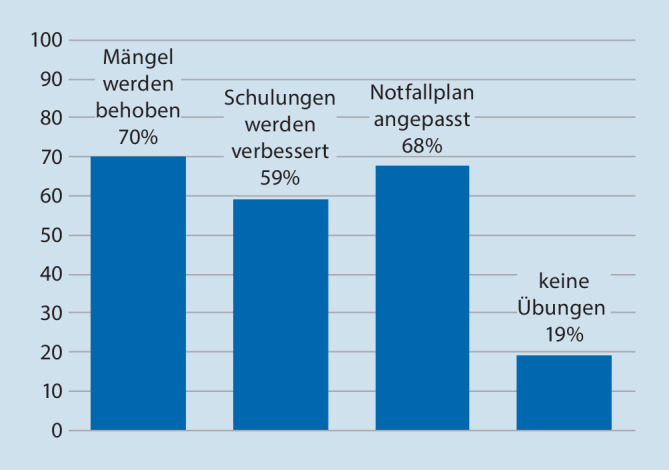

**Figure 4:**
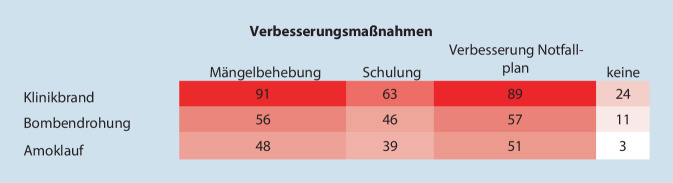

Die Kliniken, die Übungen durchführten, wurden nach Folgerungen aus diesen Übungen gefragt. Hierbei waren die Antwortmöglichkeiten „Mängel wurden behoben“, „Schulungen wurden verbessert“ und „Notfallplan wurde angepasst“ vorgegeben. Mehrfachnennungen waren möglich (Abb. 3). Bei allen angegebenen Schlussfolgerungen waren Kliniken unter 300 Betten am höchsten vertreten mit verbesserter Schulung in 17 % der Fälle, mit der Anpassung des Notfallplans in 25 % der Fälle und in 27 % mit Mängelbehebung. Kliniken über 1000 Betten sahen hingegen nur in 10 % einen Verbesserungsbedarf. Ob auch andere Folgerungen gezogen wurden, kann wegen der Fragestellung nicht angegeben werden. Am häufigsten wurden Mängelbehebungen, Verbesserung der Schulungen und Anpassungen der Notfallpläne in Hinsicht auf Brände vorgenommen, am seltensten hinsichtlich eines Amoklaufs (Abb. 4).

## Diskussion

### Auswahl der Adressaten und Repräsentanz

In der vorliegenden Arbeit sollten die Kliniken in Baden-Württemberg bezüglich ihrer Krankenhausalarm- und -einsatzplanung evaluiert werden. Im Gegensatz zu zwei früheren Befragungen in der Literatur [15, 17] wurde Wert auf eine weitgehende Kompletterfassung aller in Baden-Württemberg verzeichneten Krankenhäuser gelegt. Die Untersuchungen von Weidringer et al. [15] und Fischer et al. [17] adressierten Einzelpersonen bestimmter Fachrichtungen oder medizinischer Gesellschaften ohne weiteren Klinikbezug, sodass mehrfache oder vielfache Nennungen einzelner Kliniken (Antwort-Bias) in diesen Arbeiten nicht ausgeschlossen werden können. Von der Gesamtheit der 2014 in Baden-Württemberg gelisteten Kliniken beantworteten 63 % unsere Umfrage, sodass eine gewisse Repräsentanz gewährleistet scheint. Allerdings fällt auf, dass von den Kliniken mit weniger als 300 Betten nur knapp 44 % antworteten. Dies könnte einerseits darauf hinweisen, dass es in kleineren Häusern schwieriger ist, den richtigen Adressaten zur Befragung zu finden, andererseits wäre aber auch möglich, dass kleine Kliniken nicht antworteten, da sie keinen oder in ihren Augen keinen adäquaten KAEP besitzen.

### KAEP im internationalen Vergleich

Katastrophenvorsorge und -management bezeichnen Maßnahmen, die vor dem Eintritt einer Katastrophe getroffen werden, mit dem Ziel, zur Minimierung von Personen- und Sachschäden beim Eintritt eines solchen Ereignisses. „Damit Krankenhäuser von Großschadensereignissen oder Katastrophen nicht unvorbereitet getroffen werden, ist eine umfassende Alarm- und Einsatzplanung von entscheidender Bedeutung“ [14]. Es liegt somit im ureigensten Interesse eines Krankenhauses, Patienten, Personal und Infrastruktur durch eine entsprechende Planung vor Schäden zu bewahren [9].

Laut unserer Umfrage verfügen alle bis auf eine der antwortenden Kliniken in Baden-Württemberg über einen KAEP. Eine Studie in der Schweiz zeigte, dass von 122 Spitälern mit einer Notaufnahme etwa 82 % einen Plan zur Reaktion auf Katastrophenereignisse haben [18], dabei ergab sich ein signifikanter Unterschied zwischen privat finanzierten oder mit öffentlichen Mitteln geführten Spitälern. Eine andere Studie aus Singapur berichtete, dass 75,3 % der Beschäftigten im Gesundheitswesen glaubten, dass ihre Institution auf einen Katastrophenfall vorbereitet sei [19]. Dagegen haben frühere Studien in den USA ergeben, dass Kliniken meist weniger gut auf Katastrophen vorbereitet waren [11, 20]. Allerdings nahm nach den Anschlägen vom 11. September 2001 die Planung in den USA deutlich zu, sodass fast alle Kliniken Pläne für „chemical releases, natural disasters, epidemics and biological incidents“ vorhielten, allerdings hatten nur 80 % Pläne für Explosions- oder Brandkatastrophen [21]. In der Umfrage von Fischer et al., bei der von 7700 befragten Ärzten des TraumaNetzwerks der Deutschen Gesellschaft für Unfallchirurgie (DGU) 15,5 % antworteten, gaben 53,3 % an, dass in ihrer Klinik ein Katastrophenschutzplan vorhanden sei und sie diesen auch kennen würden [17]. Weidringer et al. eruierten in einer Umfrage im Vorfeld der Fußball-Weltmeisterschaft von 2006, dass 96 % der Antwortenden einen KAEP besäßen, allerdings hegten die Autoren wegen einer Rücklaufquote von nur 26 % an dieser Angabe selbst Zweifel [15].

Die Angabe einer Klinik, grundsätzlich einen KAEP zur Verfügung zu haben, ist ein Merkmal, das jedoch keine Aussage über Qualität und Verfügbarkeit dieses Plans zulässt. Nicht nur die Erstellung eines KAEP ist essenziell, sondern auch die Art und Weise der Bekanntgabe sowie die Einweisung des Personals. Die alleinige schriftliche Bekanntmachung ohne regelmäßige Information scheint hier nicht ausreichend zu sein [15]. Noch vor wenigen Jahren gaben deutsche Krankenhausärzte an, dass sie zwar mehrheitlich einen KAEP hätten, aber 34,3 % keine Einzelheiten kennen würden [17]. Auch in unserer Untersuchung ist der KAEP in 31 % nur in Schriftform in Sekretariaten oder Abteilungen vorhanden. Es ist somit davon auszugehen, dass auch mindestens ca. einem Drittel der Ärzte in baden-württembergischen Kliniken keine Einzelheiten des KAEP bekannt sind.

### Berücksichtigung unterschiedlicher Gefahren- und Schadenslagen

Ein traditioneller und in der Vergangenheit üblicher Ansatz bei der Erstellung eines sinnvollen KAEP war und ist es, zwischen Gefahren- und Schadenslagen zu unterscheiden, die von außen auf die Klinik zukommen (externe Lagen) oder innerhalb der Klinik auftreten (interne Lagen; [22]). Häufig werden im KAEP zwar ein Massenanfall von Verletzten sowie ein innerklinisches Brandgeschehen behandelt, technische Ausfälle der kritischen Infrastruktur „Krankenhaus“ werden hingegen deutlich seltener bedacht. So führte insbesondere der IT-Ausfall in letzter Zeit an einigen deutschen Kliniken (z. B. Düsseldorf, Köln, Wolfenbüttel, Sigmaringen) zu tage- oder monatelangen Funktionsausfällen: „Der finanzielle Schaden, der durch das Virus entsteht, ließe sich noch nicht beziffern … Aber fest steht: Jeder Tag, an dem der OP stillsteht, sorgt dafür, dass Erlöse verloren gehen“ [23]. In der vorliegenden Untersuchung gaben nur knapp 80 % der Kliniken an, dass sie sowohl externe als auch interne Schadenslagen im KAEP abbilden. Am wenigsten vorbereitet scheinen Kliniken auf einen Sauerstoffausfall zu sein. Hier muss man bedenken, dass dies auf Intensivstationen innerhalb weniger Minuten zum Tode von beatmeten, hoch sauerstoffpflichtigen Patienten führen könnte [24]. Ähnliche Ergebnisse zeigte auch eine andere Publikation, in der nur 2 % der Befragten angaben, dass sie Vorsorge für einen Sauerstoffausfall getroffen hätten [17]. In dieser Publikation findet sich auch, dass in Hinblick auf interne Schadensereignisse ein wesentlicher Unterschied zwischen Fachärzten und Nichtfachärzten besteht, so waren 61 % der Fachärzte sich ihrer Aufgaben bei internen Schadenslagen bewusst, wohingegen dies bei nur 35,3 % der Nichtfachärzte der Fall war [17].

Neben der separaten Behandlung verschiedener interner und externer Gefahrenlagen im Rahmen des KAEP [6, 9, 34] erfährt in den letzten Jahren das in seiner Grundkonzeption hiervon abweichende „konsequenzbasierte Modell“ [14, 34, 37] Beachtung. Dieser noch vergleichsweise neue Ansatz baut im Gegensatz zur traditionellem Einteilung nicht mehr auf der strikten Unterscheidung zwischen internen und externen Lagen auf, sondern basiert auf der Annahme, dass ein Gefahren- oder Schadensereignis unabhängig von seiner Ursache immer nur zwei Auswirkungen auf den klinischen Betrieb hat, nämlich die Überlastung der Versorgungskapazität (z. B. durch einen Mangel an Betten oder Personal [14]) einerseits und die Einschränkung der Funktionalität des Krankenhauses durch „technische, bauliche oder infrastrukturelle Störungen oder personelle Engpässe“ [6] andererseits. Die Vorkehrungen des KAEP sind somit stets und in erster Linie auf den „Erhalt und die Wiederherstellung der Funktionalität und der Behandlungskapazität“ [6] ausgerichtet. Hierdurch soll zum einen die Komplexität der Krankenhausalarm- und -einsatzplanung reduziert werden [14], zum anderen soll das „konsequenzbasierte Modell“ der durch die traditionelle Einteilung in externe und interne Lagen vermittelten Annahme entgegenwirken, das Krankenhaus sei lediglich für die Bewältigung von internen Gefahrenlagen selbst verantwortlich, bei externen Gefahrenlagen jedoch nicht [14, 34].

Im Rahmen der für diese Arbeit durchgeführten Umfrage gaben 26 der insgesamt 135 (19,3 %) antwortenden Krankenhäuser an, in ihrem KAEP nicht zwischen externen und internen Gefahrenlagen zu unterscheiden. Nicht untersucht wurde jedoch, ob dies real der Fall ist, d. h., ob in den betreffenden Kliniken bereits das „konsequenzbasierte Modell“ implementiert worden ist, oder ob andere Gründe für die fehlende Unterscheidung ursächlich sind.

Unsere Erhebung wurde im süddeutschen Raum – genauer im Bundesland Baden-Württemberg – durchgeführt. Ob und inwieweit die gewonnen Ergebnisse repräsentativ für ganz Deutschland sind, kann nicht beurteilt werden. Die Literatur zeigt jedoch ähnliche Entwicklungen zur sukzessiven Verbesserung der KAEP auch in anderen Bundesländern. So wird den 38 Berliner Aufnahmekrankenhäusern beispielsweise ein „Leitfaden Krankenhausalarmplanung“ als Hilfestellung für einen einheitlichen KAEP zur Verfügung gestellt. Zudem finden bereits seit 1985 in allen Berliner Aufnahmekrankenhäusern im 3‑Jahres-Turnus regelmäßige, unangekündigte „Vollübungen zur Erprobung der Einsatzbereitschaft … des Krankenhauses und der Praktikabilität der aufgestellten Einsatzpläne“ [35] statt, die im Nachgang seitens der zuständigen Senatsverwaltung und der Krankenhausleitung ausgewertet werden. Als weitere beispielhafte Maßnahmen in der Bundeshauptstadt sind die Einführung eines verpflichtenden Krankenhaus-Sichtungsalgorithmus für den MANV an allen Berliner Kliniken und die Implementierung eines Schulungskonzepts hierzu zu nennen [36]. In einigen weiteren Bundesländern existieren ebenfalls gesetzliche Verpflichtungen für Kliniken zur Mitwirkung an Übungen (z. B. in Hamburg [37], Hessen [40] oder Thüringen [41]), die teilweise auch von staatlicher Seite finanziert und ohne Ankündigung angeordnet werden. In Niedersachsen wurde im Jahr 2008 unter Beteiligung der Medizinischen Hochschule Hannover (MHH), des Deutschen Roten Kreuzes (DRK), des niedersächsischen Innenministeriums und weiterer Stellen eine Arbeitsgruppe ins Leben gerufen, die einen Leitfaden für die einheitliche Erstellung eines KAEP („Niedersächsisches Muster“) ausgearbeitet hat [38]. Im Freistaat Bayern stellt das Bayerische Staatsministerium des Innern den Krankenhäusern in Form von einheitlichen „Hinweisen für das Anlegen von Krankenhaus-Alarm- und Einsatzplänen“ Anhaltspunkte und Beispielvordrucke für die Erstellung des KAEP zur Verfügung [39]. In Thüringen wurde der Status der Krankenhausalarm- und -einsatzplanung 2017 im Rahmen einer Kleinen Anfrage des Thüringer Landtags beleuchtet. Demnach sind auch in Thüringen aufgrund des dortigen Brand- und Katastrophenschutzgesetzes die Träger der stationären Gesundheitseinrichtungen verpflichtet, „zur Mitwirkung … im Katastrophenschutz für ihre Einrichtungen Alarm- und Einsatzpläne aufzustellen und fortzuschreiben … sowie regelmäßig Übungen durchzuführen“ (§ 36 Abs. 3 ThürBKG; [41]). In 29 der 38 Thüringer Krankenhäuser (76,3 %) sind Regelungen vorhanden, die im MANV-Fall gegenseitige Unterstützung zwischen ihrem Haus und anderen Kliniken gewähren. Lediglich eine von 38 Kliniken (2,6 %) gab hingegen an, auf CBRN-Gefahrenlagen dergestalt vorbereitet zu sein, dass innerhalb von 90 min nach Alarm auf dem Klinikgelände eine Dekontaminationsstraße für die Dekontamination von Patienten vor Aufnahme in das Krankenhaus errichtet werden könnte [42].

### Schulungen und Übungen als wichtiges Instrument 

Zur Bewältigung von Gefahren- und Schadenslagen sind Ausbildung, Training und Übungen essenziell [25]. Trotzdem ist laut Literatur wenig über den Einfluss dieser Faktoren auf Planung und Vorbereitung für solche Notfallszenarien bekannt [26, 27]. Dies gilt auch für Erkenntnisse um die Auswirkungen der Planung auf das tatsächliche Management während realer Katastrophen und Großschadensereignisse [25]. Von außerklinischen Großschadensübungen ist bekannt, dass sie Mängel und Defizite sowohl in der Planung als auch in der Durchführung demaskieren können und somit dazu beitragen, eventuell ein besseres Überleben von Notfallopfern zu gewährleisten [28]. Die vorliegende Befragung ergab, dass 75 % der Kliniken Notfallübungen durchführten, wohingegen 25 % dies verneinten. Kliniken unter 300 Betten hatten mit 17 % Verneinung hier den größten Anteil. Wenn Übungen durchgeführt wurden, war dies in weniger als einem Drittel eine Vollübung; Stabsübungen wurden in 37 % durchgeführt. Vor mehr als 20 Jahren berichteten Lipp und Mitarbeiter [10], dass Katastrophenübungen in 51,2 % aller Krankenhäuser noch nie durchgeführt wurden, nur 28,4 % würden solche Übungen regelmäßig veranstalten. Weidringer et al. [15] hingegen fanden in ihrer Untersuchung zu uns ähnliche Angaben, es wurden Vollübungen in 32 % und „Planspiele“ in 46 % angegeben. Auch bei Fischer und Mitarbeitern [17] findet sich, dass die Hälfte der Kliniken mit Maximalversorgung schon eine Übung zu einem Massenanfall an Verletzten durchgeführt hat, wohingegen aber nur knapp 30 % der Grundversorger. 33,9 % der befragten Kliniken veranstalteten eine Übung zu einer internen Schadenslage [17].

Katastrophenvorsorge beinhaltet die verschiedensten Komponenten: Planung, Rüstzeug, Ausbildung, Übungen und Konsequenzen daraus. Geplante Übungen werden dabei als einer der wichtigsten Bausteine erachtet. Grundsätzlich können zwei Arten von Übungen unterschieden werden. Zum einen theoretisch gedachte Übungen, auch Stabsübungen („tabletop exercise“) genannt, zum anderen ausführende oder praktische Übungen („operation-based exercise“). Beide Arten können auch als Mischformen durchgeführt werden. Stabsübungen dienen vor allem der Überprüfung administrativ-organisatorischer Abläufe, wohingegen praktische Übungen neben der Evaluation operativ-taktischer Abläufe auch der Überprüfung gelernter Skills dienen. Praktische Übungen sind schwieriger durchzuführen und erfordern mehr Ressourcen als Stabsübungen. Sie ermöglichen jedoch das Testen von Ausrüstung, Plänen, Verfahren, Ressourcen, Technologien, behördenübergreifender Koordination und Führungseinrichtungen unter Bedingungen, die einem echten Notfallereignis sehr nahekommen [22]. Vollübungen, d. h. praktische Übungen mit stabsmäßiger Planung, führten die wenigsten Kliniken in unserer Untersuchung durch. Teilübungen, eine Übungsform bestehend aus Stabsübung und „etwas praktischer Übung“, wurden hingegen am meisten angegeben, unabhängig von der Klinikgröße. Dies ist aller Wahrscheinlichkeit nach den deutlich höheren Kosten mitgeschuldet, die eine Vollübung verursacht. Kostenintensive Übungen stehen und fallen mit der Frage nach der Finanzierung. Erst die Klärung der Finanzierungsfrage ebnet den Weg für ernsthafte Bedarfsplanungen und die Entwicklung konkret fassbarer Konzepte. Die wirtschaftliche Sicherung einer klinisch-bedarfsgerechten Versorgung der Bevölkerung ist über das Krankenhausfinanzierungsgesetz (u. a. DRG-Entgeltsystem) geregelt. Kostenintensive Katastrophenübungen sind hier nicht vorgesehen und müssen deshalb von den Kliniken selbst getragen werden. Da zudem unterschiedliche Geschäftsmodelle der Klinikbetreiber (öffentliche Trägerschaft durch Bund, Land, Landkreis oder Gemeinde; freigemeinnützige Trägerschaft durch soziale oder karitative Organisationen; private Trägerschaft mit Gewinnerzielungsabsicht) bestehen, könnte diesbezüglich eine stark unterschiedliche Motivationslage bestehen. Im Gegensatz dazu gaben in einer US-amerikanischen Studie anlässlich einer Befragung von 294 Kliniken 24,3 % an, dass sie zwischen 2002 und 2007 mehr als 150.000 US-$ an staatlicher Unterstützung zur Katastrophenvorbereitung erhalten hätten, 19,2 % erhielten mehr als 75.000 US-$ und nur 5,2 % keine Unterstützung [21].

Übungen mit realem Bezug simulieren unter Aufsicht tatsächliche Gefahren- oder Schadensereignisse. Allerdings ist bisher wenig bekannt, inwiefern eventuelle positive Erfahrungen über Jahre Bestand haben [29]. Umso wichtiger ist es, dass Übungen in regelmäßigen Abständen wiederholt werden. In der vorliegenden Erhebung gaben 61 % der Kliniken an, dass sie eine Übung in den letzten 2 Jahren durchgeführt hätten, allerdings gab auch fast ein Viertel der Antwortenden an, dass Übungen länger als 5 Jahre zurückliegen würden oder überhaupt noch nie stattgefunden hätten. Eine frühere Untersuchung im deutschsprachigen Raum ergab, dass innerhalb einer Jahresfrist ca. 50 % der Kliniken eine Übung durchführten [15]. Pikoulis und Mitarbeiter berichteten kürzlich über eine Umfrage bei 228 „health care workers“, die eine 3‑jährige Ausbildung mit 4 interdisziplinären Übungen durchliefen. Während sich vor den Übungen nur 22 % der Teilnehmer kompetent vorbereitet fühlten, stieg die gefühlte Kompetenz nach Abschluss der Übungen auf 77 % an.

Allgemein wird angenommen, dass Übungen dazu beitragen, Lücken in Notfallplänen und -verfahren zu identifizieren, die, wenn sie behoben werden, zur Verbesserung der Notfallvorsorge des Systems führen [22]. So bemerken Moss und Gaarder: „Although exercising for disaster preparedness is resource intensive, it is the repetitive, iterative nature that allows for wide staff capture and exposure along with continual improvement of plans. Having been recently involved in exercising is also likely to increase the confidence of staff and makes them feel better prepared“ [30]. Die am häufigsten in der Literatur beschriebenen Vorteile von Übungen bestehen in der Identifizierung von Lücken in bestehenden Plänen oder Protokollen [31]. In unserer Untersuchung gaben Kliniken, die Übungen durchgeführt hatten, an, dass in ca. 70 % Mängel behoben bzw. die Alarm- und Einsatzpläne angepasst wurden. An zweiter Stelle stand mit 59 % eine Intensivierung der Schulung von Mitarbeitern. Besonderer Bedarf zeigte sich hier vor allem in kleineren Kliniken, während Großkliniken weniger die Notwendigkeit zu Änderungen sahen. Der höchste „Verbesserungsbedarf“, der sich aus Übungen ergab, wurde mit 61–93 % für den Klinikbrand gesehen. Auch in der Literatur wurden anlässlich wiederholter Übungen bessere Leistungen bezüglich Brandrisiken und Sicherheitsproblemen beschrieben [32]. Inwieweit solche Verbesserungen oder Schulungen allerdings zur tatsächlichen Optimierung des KAEP in der jeweiligen Klinik beitragen, könnte nur mit einer neuerlichen Evaluation beurteilt werden. So müssen wohl die aus Übungen vorgenommenen Schlussfolgerungen mehr als „lessons identified“ denn als „lessons learned“ betrachtet werden. Auch Skryabina et al. [22] weisen auf diese Tatsache hin, denn die Autoren fanden in der Literatur keine Studien, die diesbezüglich Erfolgskontrollen durchgeführt hätten.

In den USA hat in den letzten Jahren die Entwicklung von Standards und Leitlinien für die Aus- und Weiterbildung im Katastrophenschutz an Bedeutung gewonnen. Die Notwendigkeit einer schnellen und effektiven Ausbildung des Gesundheitspersonals auf allen Ebenen wird von der Joint Commission on Accreditation of Healthcare Organizations (JCAHO) allgemein anerkannt und empfohlen [33]. Ein wirksames Krisenmanagement im Krankenhaus erfordert, dass ausgewähltes medizinisches Personal nicht nur über Wissen, sondern auch über spezifische technische Fähigkeiten und Entscheidungsfähigkeiten verfügen sollte. Ein kompetenzbasierter Ansatz könnte den Rahmen für die Durchführung dieser Art eines flexiblen Trainings bilden [14]. Das vom Bundesamt für Bevölkerungsschutz und Katastrophenhilfe herausgegebene „Handbuch Krankenhausalarm- und -einsatzplanung“ [6] skizziert zumindest einen Orientierungsrahmen zur Erstellung eines einheitlichen KAEP, eine bundesweite Verpflichtung zu einer einheitlichen Gestaltung der Planung oder gar Inhalte zu Schulung und Übungen fehlen jedoch nach wie vor.

### Einschränkungen

An der Umfrage zur Krankenhausalarm- und -einsatzplanung an baden-württembergischen Krankenhäusern haben 63 % der insgesamt bestehenden Kliniken teilgenommen. Dies erscheint uns repräsentativ für Aussagen zu sein. Allerdings ist auffällig, dass Kliniken mit weniger als 300 Betten signifikant weniger geantwortet haben. Dies könnte daran liegen, dass nicht geantwortet wurde, weil kein KAEP vorhanden ist. Damit könnte sich der prozentuale Anteil an Kliniken, die keine Alarm- und Einsatzplanung vornehmen, erhöhen.

Die dieser Arbeit zugrunde liegende Studie wurde vor Ausbruch der SARS-CoV-2-Pandemie durchgeführt. Die Häufigkeit und der Stellenwert der Vorplanungen für den Fall eines Ausbruchs allgemeingefährlicher und hochkontagiöser Infektionskrankheiten in den KAEP könnten sich durch diese Pandemie erheblich geändert haben. Auf der anderen Seite haben die Fokussierung auf die Bewältigung der Pandemie sowie Kontaktbeschränkungen in den Kliniken eventuell zu einer Reduktion an durchgeführten Übungen geführt.

Die gegebenen Antworten zu Umfragen beruhen zumeist auf der subjektiven Einschätzung des Antwortenden und nicht so sehr auf exakt erhobenen Fakten. Hierdurch können die in der Studie wiedergegebenen Ergebnisse eine gewisse Unschärfe aufweisen.

## Fazit für die Praxis

- Gegenüber früheren Untersuchungen zur Alarm- und Einsatzplanung an bundesdeutschen Kliniken zeigte unsere Untersuchung in Baden-Württemberg eine deutlich höhere Bereitschaft, eine adäquate Planung vorzunehmen wie auch hierzu Übungen durchzuführen.
- Die für Kliniken gesetzlich geforderte regelmäßige Fortschreibung und Aktualisierung des KAEP wird nur in etwas mehr als der Hälfte der Fälle innerhalb eines Jahres durchgeführt, in mehr als einem Zehntel der Kliniken vergehen sogar 3 oder mehr Jahre bis zur nächsten Aktualisierung. Hier sollte nachgebessert werden.
- Kleinere Kliniken haben Defizite an differenzierten Alarm- und Einsatzplänen, dies betrifft vor allem interne Gefahrenlagen, die durch technische Ausfälle verursacht werden.
- Fast 40 % der Kliniken führen keine Übungen zu Gefahren- und Schadenslagen durch oder die letzte Übung liegt 3 oder mehr Jahre zurück. Dies birgt die Gefahr, dass die in den Alarm- und Einsatzplänen festgelegten Verfahren nicht oder ungenügend geübt, überprüft und korrigiert werden können. Die Alarm- und Einsatzpläne sollten in regelmäßigen Abständen mit durchgeführten Übungen auf Aktualität und Validität geprüft werden.
- Bundesweite Vorgaben zur Gestaltung eines KAEP sowie zu Schulung und Übungen wären wünschenswert.
- Angesichts der weltweiten SARS-CoV-2-Pandemie sollte in weiteren Untersuchungen evaluiert werden, inwieweit sich die Vorbereitungen auf hochkontagiöse Infektionskrankheiten in den einzelnen KAEP verändert haben.

## Supplementary Information




