# Supplementary material for: Hospital disaster planning in south-western Germany. A survey of 214 clinics
Source: Notf Rett Med. 2022 Aug 16:1–10. [Article in German] Online ahead of print. doi: 10.1007/s10049-022-01065-1 (PMC9380686; doi:10.1007/s10049-022-01065-1)
Supplement: Supplementary file 1 [file 10049_2022_1065_MOESM1_ESM.pdf]

Zusatzmaterial zum Beitrag „Krankenhausalarm- und -einsatzplanung in Baden-Württemberg. Eine länderspezifische Umfrage an 214 Kliniken“ von Pfenninger EG, Villhauer S, Königsdorfer M (2022) in *Notfall+Rettungsmedizin.*
Beitrag und Zusatzmaterial stehen Ihnen auf www.springermedizin.de zur Verfügung. Bitte geben Sie dort den Beitragstitel in die Suche ein.

Fragebogen zum Thema Katastrophenplan

1. Über wie viele Betten verfügt Ihr Haus?

◯ weniger als 300 ◯ 300 – 499 ◯ 500-1000 ◯ mehr als 1000

2. Welchem Träger gehört ihr Krankenhaus an?

◯ einem privaten Träger ◯ einem staatlichen/ kommunalen Träger
◯ einem kirchlichen Träger

3. Verfügen Sie über einen Katastrophenplan / Notfallplan?

◯ Ja ◯ Nein

4. Unterscheidet der Katastrophenplan interne und externe Katastrophenlagen?

◯ Ja ◯ Nein

5. Für welche externen Lagen gibt es einen Plan?

◯ Massenanfall von verletzten Patienten
◯ Massenanfall von Verletzten bei Terroranschlag
◯ Massenanfall von Infektionen
◯ Massenanfall von intoxikierten Patienten

6. Für welche internen Lagen steht ein Notfallplan zur Verfügung?

◯ Brand ◯ Bombenbedrohung ◯ Amoklauf

7. Für welche technischen Lagen gibt es einen Notfallplan?

◯ Wasserausfall
◯ Sauerstoffausfall
◯ Heizungs- bzw. Klimaausfall
◯ Stromausfall
◯ EDV-Ausfall

8. Hat die Rettungsleitstelle Kenntnis von Ihrem Katastrophenplan?

◯ Ja ◯ Nein

9. Wird dieser Plan regelmäßig aktualisiert?

◯ Ja ◯ Nein

10. Wann wurde der Katastrophenplan zuletzt aktualisiert?

◯ vor weniger als 1 Jahr ◯ vor 1 - 2 Jahren ◯ vor 3 -5 Jahren

◯ vor mehr als 5 Jahren

11. Wie steht der Katastrophenplan den Mitarbeitern zur Verfügung?

◯ in Schriftform ausliegend, jedem zugänglich
◯ in Schriftform nur in Sekretariaten/Abteilungen
◯ im Intranet für jeden verfügbar

12. Gibt es eine personell definierte Klinikeinsatzleitung?

◯ Nein ◯ Ja

13. Werden/ wurden Notfall-/ Katastrophenübungen durchgeführt?

◯ Nein ◯ Ja als

○ Stabsübung ○ Teilübung ○ Vollübung

14. Wann wurde die letzte Übung durchgeführt?

○ vor weniger als 1 Jahr
○ vor 1-2 Jahren
○ vor 3-5 Jahren
○ vor mehr als 5 Jahren
○ Keine

15. Werden verschiedene Szenarien geübt?

○ Ja ○ Nein ○ Keine

16. Gibt es bei Ihnen Übungen, die auch externe Beteiligte (z.B. Rettungskräfte, Feuerwehr, Polizei, ABC-Zug) miteinbeziehen?

○ Ja ○ Nein ○ Keine Übungen

17. Welche Schlussfolgerungen werden aus den Übungen gezogen?

○ Mängel werden behoben
○ die Schulungen werden verbessert
○ der Notfallplan wird angepasst
○ es werden keine Übungen durchgeführt
